# Trouble Sleeping and Depression Among US Women Aged 20 to 30 Years

**DOI:** 10.5888/pcd17.190262

**Published:** 2020-04-02

**Authors:** Rifath Ara Alam Barsha, Mian B. Hossain

**Affiliations:** 1 School of Community Health and Policy, Morgan State University, Baltimore, Maryland

## Abstract

**Introduction:**

Depression in women is common, and 1 woman in 4 is likely to have an episode of major depression at some point in her life. Sleep disturbances, which are significantly associated with depression, are increasingly recognized as a determinant of women’s health and well-being. Although studies have examined the association between depression and sleep disorders, little research has explored this association among young women. Our study investigated the relationship between sleep problems and depression among women aged 20 to 30.

**Methods:**

We used data on 1,747 women from the US National Health and Nutrition Examination Survey (NHANES) 2009–2016. In addition to univariate and bivariate analysis, we used unadjusted and adjusted logistic regression models to estimate depression in the previous 2 weeks among women who reported ever having trouble sleeping.

**Results:**

Of 1,747 study participants, 19.6% reported trouble sleeping and 9.3% reported symptoms of depression. Weighted logistic regression results showed that women who had trouble sleeping were more than 4 times (odds ratio, 4.36; 95% confidence interval, 3.06–6.21; *P *< .001) more likely than women who did not have trouble sleeping to have had depression in the previous 2 weeks. The results were similar (adjusted odds ratio, 4.11; 95% confidence interval, 2.78–6.06; *P *< .001) after adjusting for other covariates.

**Conclusion:**

We found a significant relationship between trouble sleeping and depression among US women aged 20 to 30. Findings suggest the need for regular screening and treatment of sleep disturbances among young women, which may improve their psychological health and reduce depression.

**Summary**

**What is already known on this topic?**

Sleep problems are associated with depression; however, little is known about this association among young women in the United States.

**What is added by this report?**

Women aged 20 to 30 who reported having trouble sleeping were 4.1 times significantly more likely to have experienced depression in the previous 2 weeks after accounting for several covariates.

**What are the implications for public health practice?**

Regular screening and treatment of sleep disturbances are needed among US women aged 20 to 30 to reduce the prevalence of depression among this population.

## Introduction

Depression is one of the most common psychiatric disorders. In the United States, depressive disorders were the second leading cause of years lived with disability in 2010 (1). Women are more likely to have depression than men. During 2013–2016, 10.4% of US women aged 20 or older had depression in a given 2-week period and were almost twice as likely as men (5.5%) to have had depression (2). Although this sex difference persists throughout the female lifespan, it seems to vary according to reproductive stage (puberty, the week or so before menstruation, after pregnancy, and perimenopause) (3). Female hormonal fluctuation may be a trigger for depression (3). Depression is associated with decreased physical, cognitive, and social function; it is often chronic and impairs quality of life (4). Depression is predicted to be the leading cause of disease burden by 2030, and it is already the leading cause of disease burden in young adult women worldwide (3). Therefore, treatment and prevention of depression have become an important topic in the field of public health.

Sleep is an important determinant of a person’s overall health and well-being. Sleep, as a critical health-related factor, plays a role in the development of many diseases and even all-cause mortality (5). Sleep disturbance is one of the most common health complaints among young adults (6,7). Although 7 to 9 hours of sleep per night on weeknights is recommended for young and midlife adults (8), 40% of US adults have reported fewer than 7 hours of sleep per night (9). Of young adults aged 19 to 29, 67% reported not getting enough sleep to function properly (10). Many psychosocial, biological, and environmental factors contribute to insufficient sleep and sleep disturbance among young adults. The high prevalence of sleep-related disturbances may be partially due to increased academic, social, and work demands (11). Female sex is also a risk factor for sleep problems. Several studies showed that young adult women are twice as likely as young men to have poor sleep (12,13). Thus, young women appear to be a particularly vulnerable population to both sleep problems and depression.

Previous studies suggested an association between depression and sleep disturbances in older people (14,15) and an association between sleep disturbances and poor quality of life among women (16). Less research has been conducted among young adults, who are at particular risk of sleep problems and alterations in circadian timing as a result of developmental and social influences. This age group is at an important stage of life, when early interventions or treatment of sleep problems may have clinical implications. Little research has explored the relationship between sleep and depression among young women, even though the correlates of depression may differ between young women and older women. Understanding the relationships between sleep and depression and correlates among young women may increase the potential to intervene and improve mental health outcomes before they become clinically concerning. The objective of our study was to assess the relationship between trouble sleeping and depression among US women aged 20 to 30 and to determine whether trouble sleeping increased the odds of depression in this population. We hypothesized that ever having trouble sleeping would be associated with depression among women in this age group.

## Methods

We used data from the US National Health and Nutrition Examination Survey (NHANES) for 2009–2016. NHANES is a cross-sectional survey representing the noninstitutionalized civilian resident population of the United States (17). Each year, about 5,000 people are interviewed in their homes and complete the health examination components of the survey, which include medical, dental, and physiological measurements and laboratory test evaluations, usually administered in specially designed and equipped mobile examination centers located throughout the country. Interview teams consist of physicians, medical and health technicians, and trained dietary and health interviewers. The survey excludes all persons living on military bases and in institutional settings and all US citizens residing outside the 50 states and District of Columbia (17). Our analytic sample consisted of 1,747 women aged 20 to 30 who participated in NHANES at 4 sampling time points (2009–2010, 2011–2012, 2013–2014, and 2015–2016). We excluded pregnant women because pregnancy can be psychologically stressful and affect normal sleep. NHANES received approval from the National Center for Health Statistics Research Ethics Review Board, and all participants provided informed consent. Because NHANES is a public-use data set, this study was exempt from full institutional review board review. A detailed description of the data and the analytical guidelines are available elsewhere (17).

### Assessments

The sleep disorders questionnaire was administered in the home by computer-assisted personal interview during the initial survey participant interview. Participants were asked the following question about trouble sleeping: “Have you ever told a doctor or other health professional that you have trouble sleeping?” If the participant’s response to this question was yes, she was considered to have trouble sleeping.

Depressive symptoms were assessed via the Patient Health Questionnaire-9 (PHQ-9), a self-administered questionnaire that assesses depressive symptoms according to the guidelines of the fourth edition of the Diagnostic and Statistical Manual for Mental Disorders (DSM-IV) (18). Each question is scored according to the frequency of the symptom during the previous 2 weeks, and responses are made on the following scale: not at all (0), several days (1), more than half the days (2), and nearly every day (3). The PHQ-9 is considered an effective tool for assessing depressive symptoms and a reliable tool for determining instances of both subthreshold and major depression among population samples (19). A total PHQ-9 score greater than 9 (of a possible 27) indicates the presence of depression in single-screening assessments (20), and we used this cutoff. Depression was treated as the dependent variable in our analysis.

### Descriptive variables

Descriptive variables were age group (20–25 and 26–30), race/ethnicity (Hispanic, non-Hispanic white, non-Hispanic black, and other), education level (<high school graduate, high school graduate, some college, and college graduate or above), marital status (married/living together, widowed/divorced/separated, and never married), family size (1 or 2, 3 or 4, ≥5), poverty level (below 100% federal poverty level [FPL], 100%–199% FPL, 200%–299% FPL, and ≥300% FPL), health insurance (yes, has insurance vs no, does not have insurance), diabetes (yes, has diabetes vs no, does not have diabetes), and ever use of marijuana (yes vs no). The information was collected via home interview at the time of the assessments.

### Statistical analysis

NHANES uses a stratified, multistage complex survey design to enhance the representativeness of the US population. We followed the analytical guidelines suggested by NHANES. Weighting was applied to account for the sampling strata and the primary sampling unit and to adjust for oversampling and survey nonresponse. Because of the complex survey design of NHANES, we used the *svy* command in Stata version 14 (StataCorp LLP) to obtain correct variance estimation. We conducted descriptive analysis (means and proportions) of our study population. We used χ^2^ analysis to conduct bivariate analysis between all independent variables and the dependent variable (depression). Because depression is a dichotomous variable, we estimated several unadjusted and adjusted logistic regression models. We tabulated results of logistic regression models as odds ratios (ORs) and 95% confidence intervals (CIs) for all independent variables for both unadjusted and adjusted models. All percentages were reported as weighted percentages. Significance level was set at *P ≤* .05.

## Results

Approximately 9.3% of our sample of 1,747 women reported having depression in the previous 2 weeks (Table 1). The prevalence of having trouble sleeping was 19.6%. Among women who reported trouble sleeping, 22.3% reported having depression in the previous 2 weeks. Most (55.9%) of the women in our sample were aged 20 to 25. Most were non-Hispanic white (59.4%), followed by Hispanic (17.7%), non-Hispanic black (13.5%), and other races (9.4%). About two-fifths (41.9%) had some college. Most women (48.1%) were married or living together, and about 25.4% were living below 100% of the FPL. Most respondents had health insurance coverage (75.6%). More than half (57.9%) reported ever using marijuana, and marijuana use was more common among women who reported having a sleep problem (70.3%).

**Table 1:** Descriptive Statistics of the Study Sample (N = 1,747), Women Aged 20–30, National Health and Nutrition Examination Survey (NHANES), 2009–2016^a^

Variable	Trouble Sleeping		Total, n (%)
	No, n (%)	Yes, n (%)	
**Total**	1,420 (80.4)	327 (19.6)	1,747 (100.0)
**Depression in previous 2 weeks**			
No	1,327 (93.8)	253 (77.7)	1,580 (90.7)
Yes	93 (6.2)	74 (22.3)	167 (9.3)
**Age, y^b^**			
20–25	809 (57.3)	164 (50.1)	973 (55.9)
26–30	611 (42.7)	163 (49.9)	774 (44.1)
**Race/ethnicity**			
Hispanic	381 (18.7)	66 (13.4)	447 (17.7)
Non-Hispanic white	510 (57.2)	160 (68.3)	670 (59.4)
Non-Hispanic black	317 (14.3)	59 (10.5)	376 (13.5)
Other	212 (9.8)	42 (7.7)	254 (9.4)
**Education**			
<High school graduate	195 (10.6)	46 (12.2)	241 (11.0)
High school graduate	286 (18.9)	67 (21.7)	353 (19.4)
Some college	590 (41.5)	148 (43.3)	738 (41.9)
≥College graduate	349 (28.9)	66 (22.8)	415 (27.7)
**Marital status**			
Married/living together	643 (47.9)	148 (48.9)	791 (48.1)
Widowed/divorced/separated	51 (3.7)	23 (6.2)	74 (4.2)
Never married	726 (48.4)	156 (44.9)	882 (47.7)
**Family size^c^**			
1 or 2	535 (43.6)	123 (39.7)	658 (42.8)
3 or 4	522 (37.3)	131 (42.3)	653 (38.3)
≥5	363 (19.1)	73 (18.0)	436 (18.9)
**Percentage of federal poverty level**			
<100	445 (24.5)	115 (29.1)	560 (25.4)
100–199	373 (22.9)	79 (25.1)	452 (23.3)
200–299	212 (17.4)	39 (13.1)	251 (16.6)
≥300	390 (35.3)	94 (32.7)	484 (34.7)
**Has health insurance**			
No	427 (25.6)	66 (19.5)	493 (24.4)
Yes	993 (74.4)	261 (80.5)	1,254 (75.6)
**Has diabetes**			
No	1,402 (99.0)	320 (97.9)	1,722 (98.8)
Yes	18 (1.0)	7 (2.1)	25 (1.2)
**Ever used marijuana**			
No	682 (45.1)	107 (29.7)	789 (42.1)
Yes	738 (54.9)	220 (70.3)	958 (57.9)

Abbreviation: SD, standard deviation.

a Percentages and means were weighted and incorporated NHANES sample weights.

b Mean (SD) age was 24.9 (3.2) years.

c Mean (SD) family size was 3.3 (1.8).

Depression was significantly more prevalent among women who reported ever having trouble sleeping (22.6%) than among women reporting no trouble (6.5%) and significantly more prevalent among women aged 20 to 25 (10.7%) than among women aged 26 to 30 (8.1%) (Table 2). The prevalence of depression was higher among women who did not complete high school (12.9%) than among women with a high school degree (11.3%), some college (10.7%), or a college degree or above (4.1%). Similarly, the prevalence of depression was also significantly higher among women living below 100% of the FPL (12.9%) than among women in other categories of income, among women without health insurance than among women with health insurance (12.8% vs 8.3%), among women with diabetes than among women without diabetes (24.0% vs 9.3%), and among women who ever used marijuana than among women who never used it (12.9% vs 5.4%).

**Table 2:** Bivariate Relationship Between Ever Having Trouble Sleeping and Depression in Previous 2 Weeks Among Women Aged 20–30, National Health and Nutrition Examination Survey, 2009–2016

Variable	Depression in Previous 2 Weeks		χ^2^ Test (*P *Value^b^)
	No, n (%)^a^	Yes, n (%)	
**Total**	1,580 (90.7)	167 (9.3)	NA
**Ever have trouble sleeping**			
No	1,327 (93.5)	93 (6.5)	166.7 (<.001)
Yes	253 (77.4)	74 (22.6)	
**Age, y**			
20–25	869 (89.3)	104 (10.7)	13.9 (.01)
26–30	711 (91.9)	63 (8.1)	
**Race/ethnicity**			
Hispanic	412 (92.2)	35 (7.8)	13.6 (.06)
Non-Hispanic white	597 (89.1)	73 (10.9)	
Non-Hispanic black	329 (87.5)	47 (12.5)	
Other	242 (95.3)	12 (4.7)	
**Education**			
<High school graduate	210 (87.1)	31 (12.9)	56.3 (<.001)
High school graduate	313 (88.7)	40 (11.3)	
Some college	659 (89.3)	79 (10.7)	
≥College graduate	398 (95.9)	17 (4.1)	
**Marital status**			
Married/living together	733 (92.7)	58 (7.3)	31.6 (.005)
Widowed/divorced/separated	60 (81.1)	14 (18.9)	
Never married	787 (89.2)	95 (10.8)	
**Family size**			
1 or 2	600 (91.2)	58 (8.8)	7.9 (.20)
3 or 4	582 (89.1)	71 (10.9)	
≥5	398 (91.3)	38 (8.7)	
**Percentage of federal poverty level**			
<100	488 (87.1)	72 (12.9)	51.3 (<.001)
100–199	401 (88.7)	51 (11.3)	
200–299	233 (92.8)	18 (7.2)	
≥300	458 (94.6)	26 (5.4)	
**Has health insurance**			
No	430 (87.2)	63 (12.8)	21.2 (.001)
Yes	1,150 (91.7)	104 (8.3)	
**Has diabetes**			
No	1,561 (90.7)	161 (9.3)	34.3 (.001)
Yes	19 (76.0)	6 (24.0)	
**Ever used marijuana**			
No	746 (94.6)	43 (5.4)	44.1 (<.001)
Yes	834 (87.1)	124 (12.9)	

Abbreviation: NA, not applicable.

a Percentages are row percentages.

b *P* values were determined by χ^2^ test on the weighted data.

Trouble sleeping was significantly associated with depression among women aged 20 to 30 (Table 3). In the unadjusted model, women who reported trouble sleeping were more than 4 times more likely than women who did not report trouble sleeping to have had depression in the previous 2 weeks (OR, 4.36; 95% CI, 3.06–6.21; *P* < .001). The odds of depression remained high and significant (AOR, 4.11; 95% CI, 2.78–6.06; *P* < .001) among women who reported having trouble sleeping after controlling for the effect of the covariates in the model. Age was not significantly associated with depression. Education had an inverse relationship with depression: the higher the level of education, the lower the odds of depression.

**Table 3:** Odds Ratios and Confidence Intervals for the Logistic Regression Estimate for the Relationship Between Ever Trouble Sleeping and Depression in Past 2 Weeks Among Women Aged 20–30, National Health and Nutrition Examination Survey, 2009–2016

Covariate	Depression in Past 2 Weeks			
	Unadjusted Model		Adjusted Model	
	OR (95% CI)	*P* Value	AOR (95% CI)	*P* Value^a^
**Ever have trouble sleeping**				
No	1 [Reference]		1 [Reference]	
Yes	4.36 (3.06–6.21)	<.001	4.11 (2.78–6.06)	<.001
**Age, y**				
20–25	1.57 (1.09–2.26)	.01	1.39 (0.99–1.97)	.06
26–30	1 [Reference]		1 [Reference]	
**Race/ethnicity**				
Hispanic	0.74 (0.47–1.17)	.19	0.70 (0.43–1.15)	.15
Non-Hispanic white	1 [Reference]		1 [Reference]	
Non-Hispanic black	1.34 (0.91–1.98)	.13	1.08 (0.68–1.71)	.74
Other race	0.58 (0.28–1.22)	.15	0.69 (0.31–1.59)	.39
**Education**				
<High school graduate	4.14 (1.95–8.78)	<.001	2.32 (0.94–5.72)	.07
High school graduate	3.97 (1.97–8.00)	<.001	2.00 (0.93–4.29)	.08
Some college	3.24 (1.75–5.98)	<.001	1.88 (1.01–3.45)	.046
≥College graduate	1 [Reference]		1 [Reference]	
**Marital status**				
Married/living together	1 [Reference]		1 [Reference]	
Widowed/divorced/separated	2.88 (1.37–6.05)	.006	2.46 (1.08–5.62)	.03
Never married	1.77 (1.15–2.73)	.01	1.74 (1.03–2.94)	.04
**Family size**				
1 or 2	0.82 (0.51–1.29)	.38	0.89 (0.53–1.49)	.67
3 or 4	1.18 (0.75–1.84)	.47	1.17 (0.71–1.95)	.53
≥5	1 [Reference]		1 [Reference]	
**Percentage of federal poverty level**				
<100	2.89 (1.76–4.75)	<.001	1.86 (1.02–3.38)	.04
100–199	2.39 (1.37–4.15)	.003	1.67 (0.87–3.19)	.12
200–299	1.51 (0.76–3.00)	.23	1.30 (0.58–2.92)	.52
≥300	1 [Reference]		1 [Reference]	
**Has health insurance**				
No	1 [Reference]		1 [Reference]	
Yes	0.57 (0.41–0.79)	.001	0.65 (0.42–0.98)	.04
**Has diabetes**				
No	1 [Reference]		1 [Reference]	
Yes	5.62 (1.81–17.4)	.003	3.99 (1.06–15.1)	.04
**Ever used marijuana**				
No	1 [Reference]		1 [Reference]	
Yes	2.38 (1.51–3.77)	<.001	1.84 (1.09–3.12)	.02

Abbreviations: AOR, adjusted odds ratio; CI, confidence interval; OR, odds ratio.

a *P* values were determined by *t* test.

In the adjusted model, compared with women who had at least a college degree, women with some college were about 2 times more likely (AOR, 1.88; 95% CI, 1.01–3.45; *P* = .046) to have depression in the previous 2 weeks. Women who were widowed, divorced, or separated (AOR, 2.46; 95% CI, 1.08–5.62; *P* = .03) or never married (AOR, 1.74, 95% CI, 1.03–2.94; *P* = .04) were more likely to have depression than women who were married or living together. Family size was not significantly associated with depression; however, FPL was. Women living below 100% of the FPL were 86% more likely to have depression (AOR, 1.86; 95% CI, 1.02–3.38; *P* = .04) than women living at or above 300% FPL. Additionally, women with health insurance were 35% (AOR, 0.65; 95% CI, 0.42–0.98; *P* = .04) less likely to have depression than women who did not have health insurance. Having diabetes increased the odds of having depression (AOR, 3.99; 95% CI, 1.06–15.13; *P* = .04). Furthermore, ever marijuana use among women was significantly associated with depression. Women who ever used marijuana were 1.84 times (AOR, 1.84; 95% CI, 1.09–3.12; *P* = .02) more likely to have depression than women who did not use marijuana.

## Discussion

Our study demonstrated a strong and significant association between trouble sleeping and depression in a nationally representative sample of US women aged 20 to 30. Young women who reported trouble sleeping were significantly more likely to have depression than those who had no sleep problems. After adjusting for several socioeconomic, demographic, and health-related factors (ie, race/ethnicity, age, education, marital status, family size, poverty status, health insurance status, diabetes, and marijuana use), the association remained significant.

Although previous research is scant in similar populations, our findings on sleep problems among young women are consistent with those in a previous study of young women in Australia, which showed an increased risk of depression among women who reported sleep difficulties (21). Another important finding of our study is that women with lower educational attainment were more likely to report depression than those with more education; for example, women with only some college were significantly more likely than women with a college degree or more to report depression. An association between lower educational attainment and higher risk for depressive disorders was shown in previous studies. A meta-analysis found a 3% decrease in the log odds ratio for depression for each additional year of education (22). Several studies also documented the relationship between educational attainment and sleep problems: higher levels of education were associated with fewer sleep complaints (23,24). Our finding on the relationship between education and depression may be important because it strengthens evidence for the idea that obtaining higher levels of education may protect against depression among young women. Considering the possible protective role of education against depression, we further analyzed the interaction between education and sleep. However, the interaction model was not significant; hence, we did not present these findings here.

We also found a higher prevalence of depression among women living below the FPL. This finding is also congruent with previous research findings. One study reported a higher prevalence of depression in people of low socioeconomic status (25). In that study, women had a 40% greater risk of past-month depression than men, and women with income levels below the FPL had twice the risk of depression of men (25). Therefore, interventions aimed at reducing the burden of depression may need to target vulnerable populations of young women who have limited financial resources.

We found that depression was significantly associated with health insurance status and was less common among women with health insurance than among those without. This finding may be particularly important because previous research showed that uninsured people with depression and other mental illnesses were less likely than insured people to receive appropriate care (26). Furthermore, our study found that women with diabetes were significantly more likely to have depression. The prevalence of depression is higher among people with diabetes than among the general population (27). Therefore, health care providers should be aware of mental as well as physical health when treating patients with diabetes. This finding implies that health care professionals may have an important role in moderating the psychological burden of a diabetes diagnosis by addressing the patient’s psychosocial support.

Comorbidity of depression and marijuana use has been studied extensively, with evidence showing a high prevalence of depression among marijuana users (28). Our study found a higher prevalence of depression among young women who ever used marijuana than among women who had never used it, which is consistent with previous research (28). However, we could not differentiate between recreational and medical marijuana use. Given the changing political landscape of marijuana use, further studies focused on the potential effects and differences between recreational and medical marijuana use on depression among young women will be needed to shape prevention and treatment strategies.

Although our χ^2^ test found that women aged 20 to 25 were significantly more likely than women aged 26 to 30 to report depression, this relationship between age and depression was not significant after we adjusted for other covariates. That this relationship became nonsignificant may have been due to the small sample size or the effects of other factors contributing to the relationship. However, one previous study documented that the prevalence of major depression decreased with age (29). Therefore, younger age may be significantly associated with depression, and future research should further investigate the relationship between age and depression.

Our study has several strengths. Our research included a large, population-based, representative sample of young women in the assessment of the association between sleep disturbances and depression, allowing broad generalization of the results. Additionally, the NHANES data set provides information on a large sample of the general population, rather than on a specific patient group, and thereby provides information on the degree of disease burden at this level. Moreover, we made statistical adjustments for differences in a number of health and lifestyle covariates. Such an approach provided a more detailed assessment of the strength of the association.

Our study has several limitations. Although some people may have sleep disturbances before developing depressive symptoms, some may have a depression disorder before the onset of sleep disturbances. The cross-sectional design of our analysis did not allow for interpretation of the direction of the relationship, and a causal relationship cannot be inferred. Moreover, the cross-sectional design and use of secondary data limited the scope of the variable of interest. Additionally, our study estimated the prevalence of trouble sleeping among young women who ever reported having sleep disturbances to a doctor or a health professional. Therefore, we could not differentiate between women with recent sleep disturbances and women with former sleep disturbances. Another limitation of the study was that most of the information collected was self-reported. However, despite these limitations, we believe this nationally representative sample provides some insight into associations between sleep disturbance and depression among young women in the United States.

Women aged 20 to 30 with sleep disturbances should have access to screening and treatment from health professionals, and appropriate interventions should be directed to reduce depression in this vulnerable population. The association between trouble sleeping and depression has public health implications: young women seeking treatment for sleep problems may need to be screened for depression. Interventions that are designed to help such women may result in a decreased prevalence of depression in this population. Sleep is essential, and healthy sleep should be as important as healthy nutrition and physical activity in promoting overall health.
